# Adaptation and validation of the brief-satisfaction with appearance scale into Hungarian for patients with systemic sclerosis

**DOI:** 10.1186/s41687-026-01168-0

**Published:** 2026-08-11

**Authors:** Ágnes Ágoston-Szabó, Attila Szabó, Panna Pytel, Gabriella Nagy, Gábor Kumánovics

**Affiliations:** Department of Rheumatology and Immunology, Medical School, University of Pecs, Pécs, Hungary

**Keywords:** Systemic scleroderma, Quality of life, Brief-SWAP, Body image, Validation study

## Abstract

**Background:**

Systemic sclerosis (SSc) is a chronic autoimmune disease characterized by fibrosis and visible skin changes that often result in body image dissatisfaction and impaired quality of life. Despite this, appearance-related concerns remain under-investigated in SSc. The Brief Satisfaction with Appearance Scale (Brief-SWAP) is a concise, validated instrument designed to assess body image dissatisfaction and social discomfort in individuals with visible disfigurement due to SSc.

**Objective:**

To adapt and validate the Brief-SWAP scale into Hungarian using internationally accepted methodology.

**Methods:**

Sixty patients with systemic sclerosis (53 women, 7 men; mean age 57.7 ± 13.5 years; 51.7% diffuse cutaneous SSc) and 31 age- and sex-matched healthy controls (mean age 57.9 ± 14.9 years) participated in the study. The adaptation process followed the OMERACT filter methodology, including forward-backward translation and cultural adaptation. Psychometric evaluation included structural validity (via principal component analysis) reliability (internal consistency and test-retest reliability), construct validity (convergent and discriminant validity) and content validity.

**Results:**

Structural validity confirmed a two subdomains model consistent with the original structure: dissatisfaction with appearance and social discomfort. Reliability was high: internal consistency was acceptable (Cronbach’s alpha= 0.716; *p* < 0.001) and test-retest reliability was strong (ICC= 0.828). Regarding construct validity, discriminant validity was demonstrated by significantly higher Brief-SWAP scores in SSc patients versus controls (median 13.0 [IQR 6.0; 18.0] vs. 3.0 [1.0; 9.0]; *p* < 0.001) with no significant difference between limited and diffuse cutaneous SSc subgroups. Convergent validity showed moderate correlations with depression scales and SF-36 components. Content validity was confirmed, with no floor or ceiling effects observed.

**Conclusions:**

The Hungarian version of the Brief-SWAP is a valid and reliable instrument for assessing body image dissatisfaction and its psychosocial impact in patients with SSc. Its brevity and ease of use make it suitable for clinical and research applications focused on health-related quality of life.

## Introduction

Systemic sclerosis (SSc, scleroderma) is a complex autoimmune disease characterized by progressive skin fibrosis, vasculopathy, and severe internal organ involvement that drastically impairs health-related quality of life (HRQoL) [1–4]. SSc is classically divided into limited cutaneous (lcSSc) and diffuse cutaneous (dcSSc) subsets based on the extent of skin involvement, with both subgroups suffering from severe hand dysfunction and functional disability [5–6].

To accurately capture the patient’s perspective in such a heterogeneous disease, various patient-reported outcome measures (PROMs) are utilized. Organ-specific burdens, such as lung or gastrointestinal complications, are well-documented and evaluated using targeted clinical questionnaires [7–8]. Furthermore, visible changes in appearance - such as contractures and facial changes - frequently lead to severe body image dissatisfaction (BID) and psychological distress [9–13].

However, while generic instruments (e.g., Short Form 36 Health Survey - SF-36) or disease-specific tools (e.g., Systemic sclerosis Impact of Disease - ScleroID) are commonly used [14–16], they fail to capture the unique psychosocial burden of altered appearance. To address this gap, the 6-item Brief Satisfaction with Appearance Scale (Brief-SWAP) was developed to specifically evaluate BID and appearance-related social discomfort [17–18].

Currently, no PROMs focusing on appearance-related distress are available or validated in the Hungarian language. Therefore, the aim of this study was to cross-culturally adapt and validate the Hungarian version of the Brief-SWAP, filling a critical gap in patient-centered care in Hungary.

## Patients and methods

This cross-sectional, single-center observational validation study utilized a consecutive sampling method to enroll all eligible patients meeting inclusion criteria during routine clinical follow-ups between 2024 and 2025.

Sixty patients with systemic sclerosis (according to EULAR/ACR 2013 classification criteria [1]) were enrolled from the Department of Rheumatology and Immunology, University of Pecs, [53 women, 7 men, mean age 57.7 ± 13.5 years, diffuse cutaneous SSc *n* = 31 (51.7%)]. The control group included 31 [27 women, 4 men, mean age 57.9 ± 14.9 years] self-reported healthy individuals who were sex- and age-matched to the SSc patient group. In addition, being over 18 years of age were required.

Crucially, our clinical sample size (*n* = 60) satisfies the classical psychometric recommendation of a 10:1 respondent-to-item ratio (6 items x 10 = 60 patients) required to ensure stable factor analysis and robust structural validation [19].

Permission to use the brief version of Satisfaction with Appearance Scale (brief-SWAP) was given from Brett D. Thombs who developed the questionnaire with his colleagues [17]. The Brief-SWAP, derived from the original 14-item SWAP [20], consists of only 6 items. The Brief-SWAP measures the severity of self-image dissatisfaction on a 7-point Likert scale. In the clinical layout, response options are visually numbered the 1 to 7 for ease of patient completion; however, for statistical calculation and scoring, these options are coded from 0 (strongly disagree) to 6 (strongly agree).

It contains two subscales: Dissatisfaction with Appearance Scale (DAS; 3 items) and the Social Discomfort Scale (SDS; 3 items). Each question can be rated as follows: 0 = strongly disagree; 1 = disagree; 2 = somewhat disagree; 3 = neutral; 4 = somewhat agree; 5 = agree; and 6 = strongly agree. Specifically, the items evaluate distinct cosmetic and social dimensions, including satisfaction with facial features (Item 1), the appearance of limbs and hands (Items 2 and 3), social anxiety in public settings (Item 4), perceived lack of attractiveness to others (Item 5), and the fear of social or physical rejection (Item 6). The score for each subscale ranges from 0 to 18, calculated by summing the respective three items. The total Brief-SWAP score is the sum of all six items, ranging from 0 to 36, where higher scores indicate greater body image dissatisfaction and appearance-related distress [17, 21]. 

Subjects completed the full questionnaire packet (including reference scales the Patient Health Questionnaire-9 (PHQ-9) [22], Beck Depression Questionnaire (Beck-D) [23], Center for Epidemiologic Studies Depression Scale (CES-D) [24] and the Short Form 36 (SF-36) Healthy Survey [14, 16]) on Day 1 to establish cross-sectional validity. To evaluate test-retest reliability, patients re-administered only the Brief-SWAP scale on Day 5, assuming their clinical status remained stable over this brief 5-day window.

The reference questionnaires - SF-36, PHQ-9, Beck-D, and CES-D - were chosen because they represent gold-standard instruments for measuring generic physical/mental health-related quality of life and varying severity levels of depressive symptomatology, which are known to clinically overlap with body image distress. Furthermore, the selection of these specific reference scales directly aligns with the methodology implemented in the original validation study of the Brief-SWAP, ensuring international comparability of our concurrent validity analyses [17]. 

In addition, clinical data were also assessed - such as patient history, physical examination, patient’s therapy, Disease Activity Score 28 (DAS-28) [25], laboratory (antibodies and basic laboratory), respiratory function and cardiac ultrasound, telangiectasia score [26], modified Rodnan skin score (mRSS) [27], manual muscle strength test (MMT8) [28], hand function (Hand Anatomy Index, HAI) [29], Mouth Opening Ability Assessment (MOA) [30] and Medical and Patient Visual Analogue Scales (VAS) [31]. For analyses of topographical skin involvement, only evaluable site-specific mRSS assessments were included; percentages were calculated based on available data.

### Validation of brief-SWAP in Hungarian

The validation and adaptation of Brief-SWAP followed the validation methodology recommendation for rheumatological disorders called “OMERACT filter” [32] and the COSMIN guidelines for systematic reviews of patient-reported outcome measures [33]. The 6 questions of the questionnaire and their answer options were translated from English to Hungarian and adapted to the Hungarian culture by the “back and forward translation method”. First, a physician, a healthcare professional and a patient with SSc translated the questionnaire from English into Hungarian. Then the expert group, together with another physician, created the questionnaire in Hungarian based on a consensus opinion. Subsequently, an English language teacher and a native Hungarian-speaking health worker living in an English-speaking area translated the Hungarian questionnaire back into English. There were no significant differences between the original and the translated questionnaire. During the forward-backward translation and pilot phase, no items required cultural modification or idiomatic restructuring, as the aesthetic and social concepts regarding facial, hand, and skin changes were found to be fully transparent and equivalent in the Hungarian healthcare and cultural context [34]. The expert group finalised the Brief-SWAP in Hungarian. (Annex 1)

## Annex 1

### Hungarian version of the Brief-SWAP questionnaire

**Table:** 

	Egyáltalán nem értek egyet	2	3	4	5	6	Teljes mértékben egyetértek
**Arc vonások**							
1. Elégedett vagyok az arcom megjelenésével.							
**Egyéb testrészek megjelenése**							
2. Elégedett vagyok a kezeim megjelenésével.							
3. Elégedett vagyok a karjaim megjelenésével.							
**Szociális szorongás**							
4. Kényelmetlenül érzem magam idegenek társaságában amiatt, ahogy a kinézetem megváltozott a scleroderma betegségtől.							
**Érzékelt társadalmi hatás**							
5. Úgy érzem, hogy a sclerodermám nem vonzó mások számára.							
6. Nem hiszem, hogy az emberek meg akarnának érinteni.							

A trial version of the questionnaire was tested on a representative sample (10 people)

### Measures and statistical analysis

The psychometric and statistical properties of the scale were analysed comprehensively in a single logical sequence. Structural validity was examined using principal component analysis to confirm the expected two-subdomain distribution. Reliability was assessed via internal consistency using Cronbach’s alpha, and test-retest reliability was evaluated by re-administering the scale on Day 5 (assuming a stable clinical status over this 5-day interval), calculating the intraclass correlation coefficient (ICC). Construct validity comprised convergent validity - evaluated by Spearman’s rank correlation with related PROMs (PHQ-9, Beck-D, CES-D, and SF-36) - and discriminant validity. To evaluate discriminant validity, the Mann-Whitney U test was used for direct two-group comparisons between the total SSc cohort and healthy controls. Additionally, to investigate variations across clinical subsets and healthy volunteers, multi-group comparisons were performed using the non-parametric omnibus Kruskal - Wallis test, with subsequent post-hoc pairwise comparisons adjusted via Bonferroni correction. Content validity was evaluated by analysing floor and ceiling effects, where a threshold of ≤ 15% of patients achieving the absolute minimum or maximum score was considered acceptable to rule out significant effects, in accordance with established quality criteria [35]. Due to the non-normal distribution of the data, continuous variables are expressed as median (25–75% interquartile range [IQR]).

All statistical analyses were performed using IBM SPSS Statistics Version 29.0.2.0 software. All subjects gave written informed consent prior to study enrollment, following a comprehensive verbal and written explanation of the protocol. The study was authorized by the Scientific and Research Ethics Committee (registration number: ETT-TUKEB BM/29196-1/2023) and conducted in strict accordance with the Declaration of Helsinki.

## Results

### Clinical characteristics

The demographic and clinical characteristics of the study population are summarized in Table 1. The mean age of the 60 included systemic sclerosis (SSc) patients was 57.7 ± 13.5 years, and the cohort was predominantly female (*n* = 53, 88.3%). The mean disease duration was 11.7 years ± 7.0. Regarding organ involvement gastrointestinal manifestation was observed in 31 patients (51.7%) and pulmonary fibrosis detected by HRCT was present in 42 subjects (70.0%). Pulmonary arterial hypertension (PAH) confirmed by right heart catheterization was present in 4 patients (6.7%), while cardiac involvement was observed in 30 subjects (50%). No patients had renal involvement. In terms of autoantibody profiles, antinuclear antibody (ANA) positivity was detected in 85% (*n* = 51) of the included patients, anticentromere antibody positivity (ACA) in 18.3% (*n* = 11), and anti-Scl-70 antibody positivity in 36.7% (*n* = 22). RNA polymerase antibody positivity was observed in 6.7% (*n* = 4) of the cohort.

**Table 1:** Clinical characteristics of the systemic sclerosis patient group

Variable	Systemic sclerosis patients (*n* = 60)	Limited cutaneous systemic sclerosis patients (*n* = 29)	Diffuse cutaneous systemic sclerosis patients (*n* = 31)
Age (years, mean ± SD)	57.7 ± 13.5	58.2 ± 15.0	57.1 ± 12.2
Gender (female, male, n)	53/7	26/3	27/4
Disease duration (years, mean ± SD)	11.7 ± 7.0	11.2 ± 7.8	12.0 ± 6.4
Gastrointestinal involvement n (%)	31 (51.7)	13 (44.8)	18 (58.1)
Pulmonary fibrosis by HRCT n (%)	42 (70.0)	17 (58.6)	25 (80.6)
Pulmonary Arterial Hypertension n (%)	4 (6.7)	1 (3.4)	3 (9.7)
Cardiac involvement n (%)	30 (50.0)	16 (55.2)	14 (45.2)
Renal involvement n (%)	0 (0.0)	0 (0.0)	0 (0.0)
Antinuclear Antibody positivity n (%)	51 (85.0)	26 (89.7)	25 (80.6)
Anticentromere Antibody positivity n (%)	11 (18.3)	10 (34.5)	1 (3.2) *
Anti-Topoisomerase I Antibody positivity n (%)	22 (36.7)	9 (31.0)	13 (41.9)
RNA polymerase III positivity n (%)	4 (6.7)	1 (3.4)	3 (9.7)
Prevalence of small joint contractures n (%)	22 (36.7)	7 (24.1)	15 (48.4) *
Subcutaneous calcinosis n (%)	12 (20.0)	5 (17.2)	7 (22.6)
Nr of shortened fingers (%)	8 (13.3)	4 (13.8)	4 (12.9)
Prevalence of telangiectasia n (%)	56 (93.3)	26 (89.7)	30 (96.8)
modified Rodnan skin score (median, [IQR]) ^	7.0 (3.3; 9.8)	6.0 (3.0; 8.0)	9.0 (5.0; 11.0) *
Health Assessment Questionnaire score (median, [IQR])	0.4 (0.0; 1.3)	0.3 (0.0; 0.9)	0.5 (0.1; 1.4)
Short Form 36 Health Survey, Physical Component Summary score (median, [IQR])	70.0 (50.6; 85.0)	74.4 (51.2; 86.8)	66.9 (49.4; 85.0)

* Significant difference between limited and diffuse cutaneous disease groups (*p* < 0.05)

SD: Standard Deviation; IQR: Interquartile Range; HRCT: High-Resolution Computed Tomography; SSc: Systemic Sclerosis; SF-36: Short Form-36

^ Data missing for 11 patients; calculated from *n* = 49 patients

### Musculoskeletal and cutaneous features

Within the musculoskeletal and cutaneous domain small joint contractures of the hand were present in 36.7% (*n* = 22) of the patients. Subcutaneous calcinosis occurred in 12 patients (20%), while shortened fingers were present in 8 patients (13.3%). Telangiectasia was shown in 56 patients (93.3%) in the study group. The overall median modified Rodnan skin score (mRSS) was 7.0 (IQR 3.3; 9.8). Topographical analyses were based on available mRSS assessments (24/31 dcSSc and 25/29 lcSSc patients). Anatomical mapping via the mRSS revealed that facial skin involvement was present in 43/49 patients (87.8%), finger involvement in 49/49 patients (100.0%), hand involvement in 32/49 patients (65.3%), and forearm involvement in 19/49 patients (38.8%).

When comparing the clinical characteristics of the diffuse and limited cutaneous systemic sclerosis groups, significant differences were observed in the prevalence of ACA positivity, the frequency of joint contractures and the extent of skin involvement. In contrast, no significant intergroup differences were detected with respect to other cutaneous manifestations. (Table 1)

### Psychometric evaluation of the scale

During the cross-cultural adaptation phase, a pilot version of the translated instrument was administered to 10 consecutive SSc patients, independent of the main study cohort. No comprehension issues or structural modifications were suggested. The average completion time of the Brief-SWAP was 1.0 min.

See Table 2 for the results of the Brief-SWAP and the reference questionnaires used for validation.

**Table 2:** Brief-SWAP total, subscales and the reference questionnaires scores

Questionnaire / Scale (median [IQR])	Systemic sclerosis patients (*n* = 60)^10^	Limited cutaneous systemic sclerosis patients (*n* = 29)^11^	Diffuse cutaneous systemic sclerosis patients (*n* = 31)^11^	Healthy volunteers (*n* = 31)^11^
Brief-SWAP^1^ score	13.0 (6.3; 18.0) *	12.0 (5.0; 18.0) ^a^	14.0 (7.0; 18.0) ^a^	3.0 (1.0; 9.0)
Brief-SWAP DAS^2^ score	8.0 (3.3; 12.0) *	7.0 (3.0; 10.5) ^a^	9.0 (4.0; 13.0) ^a^	3.0 (1.0; 6.0)
Brief-SWAP SDS^3^ score	3.0 (0.0; 8.8) *	3.0 (0.0; 8.5) ^a^	3.0 (0.0; 9.0) ^a^	0.0 (0.0; 0.0)
Beck-D^4^ score	3.0 (1.0; 6.0) *	3.0 (1.0; 6.0)	3.0 (2.0; 4.0)	1.0 (0.0; 4.0)
PHQ-9^5^ score	4.5 (2.0; 7.0) *	5 (2.0; 8.5)	4.0 (1.0; 7.0) ^a^	2.0 (0.0; 4.0)
CES-D^6^ score	8.0 (3.0; 15.8)	9.0 (3.5; 15.5)	8.0 (3.0; 19.0)	5.0 (1.0; 11.0)
SF-36^7^ PCS^8^ score	70.0 (50.6; 85.0) *	74.4 (51.2; 86.9)	66.9 (49.4; 85.0)	87.5 (62.5; 92.5)
SF-36 MCS^9^ score	85.4 (61.9; 94.3)	84.9 (59.6; 94.3)	85.5 (62.3; 94.3)	86.5 (71.8; 97.8)

^1^Brief-Satisfaction With Appearance Scale, ^2^Dissatisfaction with Appearance Subscale, ^3^Social Discomfort Subscale, ^4^Beck Depression Scale, ^5^Patient Health Questionnaire-9, ^6^Center for Epidemiologic Studies Depression Scale, ^7^Short Form 36 Health Survey ^8^Physical Component Summery, ^9^Mental Component Summery, IQR: interquartile range, ^10^ Compared using Mann-Whitney U test (Total SSc vs. Healthy volunteers), ^11^Compared using Kruskal-Wallis test with subsequent pairwise comparisons (Bonferroni-corrected)

* Significant difference between Total SSc patients and healthy volunteers (*p* < 0.05)

ª Significant difference compared specifically to healthy volunteers based on pairwise comparisons (*p* < 0.05)

Note: No statistically significant differences were found between lcSSc and dcSSc subgroups on any of the scales (*p* = 1.000)

#### Structural validity

Using principal component analysis, questions were grouped into two main components. Questions 1, 2, and 3 were included in the first dimension (Dissatisfaction with Appearance), while questions 4, 5, and 6 were included in the second dimension (Social Discomfort).

#### Reliability

The internal consistency of the Hungarian Brief-SWAP was acceptable, with a Cronbach’s alpha of 0.716 (*p* < 0.001). To assess test - retest reliability, patients completed the questionnaire twice, at enrollment and 5 days later. The ICC between the scores of the two completions was found to be 0.828 (*p* < 0.001), demonstrating high stability over time.

#### Construct validity

##### Convergent validity

In the test of convergent validity, using Spearman’s rank correlation analysis, a significant correlation was found for all questionnaires tested, including diffuse and limited cutaneous subgroups, except for the PHQ-9 for diffuse cutaneous scleroderma and for the SF-36 PCS for limited cutaneous scleroderma (lcSSc) (Table 3).

**Table 3:** Correlation analysis of Brief-SWAP and reference questionnaires

	Systemic sclerosis patients (*n* = 60)		Limited cutaneous systemic sclerosis patients (*n* = 29)		Diffuse cutaneous systemic sclerosis patients (*n* = 31)	
	Rho	*p*	Rho	*p*	Rho	*p*
Beck-D^1^	0.445	< 0.001	0.478	0.007	0.479	0.010
PHQ-9^2^	0.288	0.025	0.218	0.240	0.432	0.019
CES-D^3^	0.441	< 0.001	0.459	0.009	0.452	0.014
SF-36^4^ PCS^5^	− 0.358	0.005	− 0.250	0.175	− 0.481	0.010
SF-36 MCS^6^	− 0.421	< 0.001	− 0.432	0.015	− 0.466	0.012

^1^Beck Depression Scale, ^2^ Patient Health Questionnaire-9, ^3^Center for Epidemiologic Studies Depression Scale ^4^Short Form 36 Health Survey ^5^Physical Component Summery ^6^Mental Component Summery

##### Discriminant validity

**Fig. 1:**
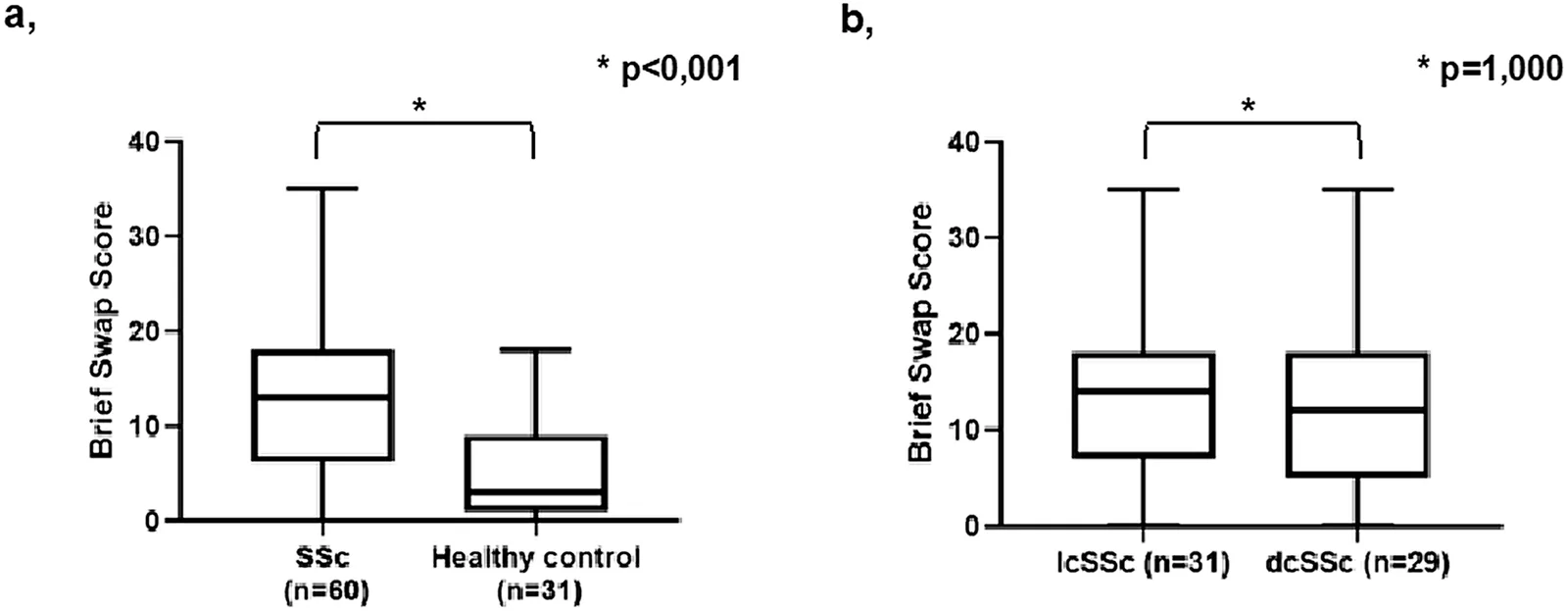
Discriminant validity test comparing (**a**) healthy and scleroderma groups (**b**) patients with lcSSc and dcSSc

To evaluate the discriminative validity of the Hungarian version of the Brief-SWAP and the reference scales, scores were compared between SSc patients and healthy volunteers using the Mann-Whitney U test, and across the three study groups using the non-parametric Kruskal-Wallis test to identify distinct clinical patterns between the disease subsets and healthy volunteers (Table 2). Using the Mann-Whitney U test, SSc patients scored significantly higher on the total Brief-SWAP score than healthy controls (median 13.0 [IQR 6.0; 18.0] vs. 3.0 [1.0; 9.0]; *p* < 0.001) (Fig. 1a). For the multi-group comparison, the Kruskal-Wallis test revealed significant overall differences among the groups across both subscales and the total score: Dissatisfaction with Appearance Scale (DAS) (H(2) = 15.7, *p* < 0.001), Social Discomfort Scale (SDS) (H(2) = 21.1, *p* < 0.001), and the Brief-SWAP total score (H(2) = 18.9, *p* < 0.001). Subsequent pairwise comparisons with Bonferroni correction demonstrated that the scale possesses an excellent and unique capacity to clearly differentiate both the limited cutaneous SSc (lcSSc) and diffuse cutaneous SSc (dcSSc) subgroups individually from healthy controls (adjusted *p* < 0.05).

In contrast, the generic psychological and quality of life screeners showed a much more overlapping and less discriminative pattern across subsets. For depression severity measured by the PHQ-9, only the limited SSc (lcSSc) subgroup differed significantly from healthy individuals (adjusted *p* = 0.011), while the diffuse SSc (dcSSc) subgroup did not. Furthermore, no statistically significant pairwise differences could be established between either of the individual SSc subgroups and healthy volunteers regarding the Beck-D, the CES-D, or the SF-36 Physical (PCS) and Mental (MCS) Component Summaries (*p* ≥ 0.05). Conversely, direct pairwise comparisons between the disease subgroups robustly confirmed that there were no statistically significant differences on any of the investigated scales; the lcSSc subgroup and the dcSSc subgroup exhibited nearly identical levels of body image dissatisfaction and generic distress, yielding a perfect non-significant adjusted p-value of 1.000 across all subscales and total indices. This statistical equivalence between the lcSSc and dcSSc subgroups is illustrated in Table 2; Fig. 1b.

#### Content validity

When testing the “floor-ceiling” effect, 5% of patients reached the minimum score, but this did not exceed the acceptable 15% threshold. No patients achieved the maximum score, indicating appropriate scale targeting for this patient population.

## Discussion

The Brief-SWAP was successfully adapted and validated for use in Hungarian in accordance with international methodological standards, including the OMERACT filter [32]. The translation and back-translation process involved both healthcare professionals and patients, and no modifications were required following pilot testing. The scale proved brief, user-friendly, and feasible for routine clinical practice, with an average completion time of less than one minute.

Compared with the original validation study by Jewett et al. [17], several demographic and clinical differences were observed. The original sample was larger and consisted exclusively of women, whereas our cohort included both genders. Ethnic composition is also different (current research: 100% vs. 89.9% white), and our participants had a slightly longer disease duration (current research: 11.7 ± 7.0 vs. 8.4 ± 9.2) and a lower proportion of university (current study: 26.7% vs. 47.7%) graduates. Despite these differences, the psychometric performance of the Hungarian version closely aligned with that of the original tools (original scores: 13.6 ± 8.4 and 13.5 ± 8.8; current research: 13.0 ± 8.3).

In terms of structural validity, our principal component analysis successfully replicated the two-subdomain structure of the original scale developed by Jewett and colleagues. The items cleanly loaded onto two distinct factors: the Dissatisfaction with Appearance Scale (DAS) and the Social Discomfort Scale (SDS). This structural robustness confirms that the Hungarian version measures the exact same underlying psychological dimensions as the original English version.

The reliability of the Hungarian version proved to be highly satisfactory. Internal consistency was acceptable with a Cronbach’s alpha of 0.716, which fully satisfies the standard psychometric threshold of > 0.70 for established scales [35]. Furthermore, test - retest reliability was exceptionally strong, yielding an ICC of 0.828. This exceeds the recommended criterion of > 0.70 for stable instruments and confirms that the scale provides highly reproducible results over time when the clinical status remains unchanged.

Our evaluation of construct validity provided compelling evidence for both the discriminant and convergent validity of the scale. Regarding discriminant validity, the scale demonstrated an excellent capacity to differentiate SSc patients from healthy controls, with patients scoring significantly higher on both the total scale and subscales (median 13.0 vs. 3.0; *p* < 0.001). In our study, a noteworthy pattern emerged regarding generic psychological measures. While the Beck-D and CES-D scales did not demonstrate significant overall group differences, the PHQ-9 identified significantly higher depressive symptoms specifically in the lcSSc subgroup compared to healthy controls (adjusted *p* = 0.011). This finding is particularly interesting given that diffuse cutaneous involvement is traditionally associated with higher physical and skin disease activity. However, several studies in the SSc literature suggest that patients with the limited subset often experience profound psychological distress due to the slowly progressive but highly disfiguring nature of hand and facial changes, which directly impacts daily functioning, self-identity, and social interactions [10, 17, 23]. This emphasizes that generic instruments like the PHQ-9 capture broad affective and vegetative distress that, in lcSSc, tracks heavily with the psychological toll of chronic, localized disease progression [23]. Conversely, CES-D and SF-36 MCS scores did not differ significantly between groups, which may reflect the multifactorial nature of depression and mental health. By specifically assessing body image and appearance-related social discomfort, the Brief-SWAP appeared more sensitive to the psychosocial impact of SSc.

Regarding disease subtypes, our cohort contained relatively fewer patients (29 persons / 48.3% vs. 472 persons / 72.2%) with limited cutaneous SSc than the original validation study; however, we found no significant difference in Brief-SWAP total scores between the limited and diffuse groups. This is notable because, to our knowledge, no previous study has compared total Brief-SWAP scores across SSc subtypes. Earlier research examined only the two subscales (Dissatisfaction with Appearance and Social Discomfort) of the Brief-SWAP. In those studies, including those by Mills et al. and Fox et al., patients with diffuse disease consistently exhibited higher subscale scores. For example, diffuse patients scored higher on the Dissatisfaction with Appearance subscale (dcSSc 11.2 ± 4.5 vs. lcSSc 8.2 ± 5.1) and on the Social Discomfort subscale (dcSSc 8.1 ± 5.1 vs. lcSSc 5.3 ± 5.1) compared with limited patients. This absolute absence of phenotypic variance in our cohort was robustly confirmed by our post-hoc Bonferroni-adjusted pairwise analysis, which yielded a perfect non-significant adjustment (*p* = 1.000) between the dcSSc and lcSSc subgroups across all subscales and total scores.

In our sample, this statistical identity and the homogeneous distribution across clinical subsets may reflect the comparable localized severity of skin involvement in socially prominent areas rather than the overall systemic skin burden. Unlike global psychological distress, body image dissatisfaction tracks directly with visible physical disfigurement. Patients with limited SSc suffer from severe, highly apparent changes fundamentally localized to the face (microstomia, telangiectasias) and the hands (acrosclerosis, flexion contractures). Because these anatomical regions are central to social communication and self-identity, the appearance-related distress is just as profound in lcSSc as it is in the diffuse form. Importantly, our analysis also emphasized that while the two disease subtypes do not differ from each other, both the lcSSc and dcSSc subgroups exhibit drastically and significantly higher appearance-related distress compared to healthy controls (*p* < 0.001). This underscores that the psychological and psychosocial impact of visible disfigurement is profound and universal across the entire SSc spectrum, regardless of the clinical subset. Although patients with diffuse cutaneous SSc exhibited a significantly higher total modified Rodnan skin score than patients with limited cutaneous SSc (median 9.0 vs. 6.0, *p* < 0.05), involvement of highly visible anatomical areas remained frequent in both subsets. Facial involvement was observed in 83.3% of evaluable dcSSc patients and 92.0% of evaluable lcSSc patients, while involvement of the hand region was present in nearly all evaluable patients. Because the Brief-SWAP specifically targets appearance satisfaction related to the face, hands, and arms, skin changes in these socially prominent areas may have a greater impact on appearance-related distress than the overall extent of skin involvement, potentially explaining why Brief-SWAP scores did not differ significantly between the lcSSc and dcSSc subgroups.

Finally, convergent validity was confirmed by the significant correlations observed between the Brief-SWAP and standard health-related quality of life and depression measures. The correlations between the Brief-SWAP and global psychological measures (such as the PHQ-9 or Beck-D) were moderate (*rho* = 0.288 to 0.445). This low-to-moderate correlation is conceptually expected and further supports the construct validity of the scale. While depression scales capture broad, generic affective and vegetative distress, the Brief-SWAP evaluates a highly specific construct: body image dissatisfaction secondary to visible disfigurement. These scales should not heavily correlate, proving that the Brief-SWAP captures unique clinical dimensions that standard psychiatric screeners miss, in direct alignment with the findings of the original validation study.

Regarding content validity, the analysis of floor and ceiling effects demonstrated that the scale is well-targeted for patients with systemic sclerosis. The floor effect (5.0%) was well below the conservative 15% threshold, and no ceiling effect was observed, ensuring that the instrument can accurately capture changes in appearance-related distress across the entire spectrum of disease severity.

To our knowledge, this study provided the first validated non-English version of the Brief-SWAP. Although a Thai translation has been mentioned in the literature, no formal validation procedures were reported [21]. Additional cross-cultural validations would further strengthen the international applicability of the instrument.

### Clinical implications

Our findings indicated that patients with lcSSc experienced appearance-related distress comparable to those with diffuse disease, highlighting the importance of providing equal psychosocial attention to both subtypes. Given the involvement of prominently visible areas such as the face and hands, clinicians should be aware that appearance-related concerns may be substantial even in patients traditionally regarded as having milder disease. In addition, the use of this questionnaire better reflects the decline in quality of life caused by external factors than general questionnaires.

### Limitations

This study was conducted at a single center with a smaller sample size than the original validation study, and the distribution of participants across early and late disease stages was unbalanced. In addition, men represented only 10% of the sample, limiting generalizability to male patients. Nonetheless, these limitations did not compromise the overall psychometric conclusions.

Importantly, the Department of Rheumatology and Immunology at the University of Pécs serves as a national tertiary reference center for systemic sclerosis, drawing a highly representative spectrum of patients from a large geographic region. Given that systemic sclerosis is a severe disease with well-documented clinical outcomes and specific survival characteristics among Hungarian patients [36], utilizing data from such a major national cohort strongly enhances the relevance of our findings. Furthermore, our department is an active member of the European Scleroderma Trials and Research group (EUSTAR) network, holding a GOLD certification for clinical registry compliance. which ensures that patient data frequently undergo strict international monitoring with excellent results.

## Conclusions

In summary, the Hungarian adaptation of the Brief-SWAP demonstrated satisfactory validity, reliability, and usability. It offered an efficient means of assessing appearance-related distress and social discomfort in patients with systemic sclerosis and provided valuable psychosocial insight that complemented traditional clinical measures.

## Data Availability

The datasets supporting the conclusions of this article are included within the article. All original documentation generated and analyzed during the current study are available from the corresponding author on reasonable request.

## Abbreviations

SSc: Systemic sclerosis, scleroderma
Brief-SWAP: Brief Satisfaction with Appearance Scale
OMERACT: Outcome Measurement in Rheumatology
COSMIN: COnsensus-based Standards for the selection of health Measurement Instruments
PHQ-9: Patient Health Questionnaire-9
Beck-D: Beck Depression Scale
CES-D: Center for Epidemiologic Studies Depression Scale
SF-36: Short Form 36 Health Survey
PCS: Physical Component Summery
MCS: Mental Component Summery
ICC: Inter Correlation Consistency
QoL: Quality of Life
HRQoL: Health-Related Quality of Life
SleroID: Systemic sclerosis Impact of Disease
BID: Body Image Dissatisfaction
DAS: Dissatisfaction with Appearance Scale
SDS: Social Discomfort Scale
SWAP: Satisfaction with Appearance Scale
PROM: Patient Reported Outcome Measures
EULAR/ACR: European Alliance of Associations for Rheumatology/American College of Rheumatology
DAS-28: Disease Activity Score 28
mRSS: Modified Rodnan Skin Score
MMT8: Manual muscle strength test 8
HAI: Hand Anatomy Index
MOA: Mouth Opening Ability Assessment
VAS: Visual Analogue Scales
HRCT: High-Resolution Computed Tomography
PAH: Pulmonary arterial hypertension
ANA: Antinuclear antibody
ACA: Anticentromere antibody positivity
lcSSc: Limited cutan systemic sclerosis
dcSSc: Diffuse cutan systemic sclerosis
HAQ: Health Assessment Questionnaire
